# Supplementary material for: Human Papillomavirus Infection and the Risk of Erectile Dysfunction: A Nationwide Population-Based Matched Cohort Study
Source: J Pers Med. 2022 Apr 27;12(5):699. doi: 10.3390/jpm12050699 (PMC9145882; doi:10.3390/jpm12050699)
Supplement: Supplementary file 1 [file jpm-12-00699-s001.zip › jpm-1625710-supplementary.pdf]

Supplement Table S1. Baseline characteristic in sensitivity analysis (excluding participants with cancer and stroke on the index date)

	Non-HPV (N=46708)		HPV (N=11677)		
Variables	n	%	n	%	SMD
Age, year					
18-30	19217	41%	4784	41%	0.004
31-40	10367	22%	2554	22%	0.008
41-50	8907	19%	2173	19%	0.01
>=51	8217	18%	2166	19%	0.03
mean, (SD)	36.6	(13.9)	36.8	(14.4)	0.02
Comorbidities					
Hypertension	6330	14%	1603	14%	0.005
diabetes mellitus	2897	6%	763	7%	0.01
Hyperlipidemia	6948	15%	1740	15%	0.001
CAD	296	0.6%	99	0.8%	0.03
CKD	235	0.5%	91	0.8%	0.04
COPD	3232	6.9%	881	7.5%	0.02
Alcohol-related illness	1426	3.1%	365	3.1%	0.004
HIV	104	0.2%	30	0.3%	0.007
Medication a-blocker					
	4030	9%	1143	10%	0.04
b-blocker	2310	5%	628	5%	0.02
CCB	6178	13%	1587	14%	0.01
Diuretics	3708	8%	978	8%	0.02

SMD: standard mean difference;

CAD: cardiovascular disease; CKD: chronic kidney disease; COPD: chronic obstructive pulmonary disease;

HIV: human immunodeficiency virus; AID: Autoimmune disease; CCB: calcium channel blocker.

Supplement Table S2. Sensitivity analysis (excluding participants with cancer and stroke on the index date)

	Erectile dysfunction						
Variables	n	PY	IR	cHR	(95% CI)	aHR ǂ	(95% CI)
non-HPV	332	251972	1.32	1.00	-	1.00	-
HPV	135	63016	2.14	1.64	(1.34,2)***	1.59	(1.3,1.95)***
Age, year							
18-30	57	136895	0.42	1.00	-	1.00	-

31-40	80	70296	1.14	2.74	(1.95,3.86)***	2.41	(1.71,3.39)***
41-50	148	58493	2.53	6.12	(4.51,8.31)***	4.16	(3.02,5.72)***
>=51	182	49304	3.69	9.03	(6.7,12.16)***	3.90	(2.75,5.53)***
Comorbidities							
hypertension							
No	344	276908	1.24	1.00	-	1.00	-
Yes	123	38080	3.23	2.62	(2.13,3.22)***	0.84	(0.63,1.12)
diabetes mellitus							
No	389	297492	1.31	1.00	-	1.00	-
Yes	78	17496	4.46	3.43	(2.69,4.37)***	1.27	(0.97,1.67)
hyperlipidemia							
No	301	271359	1.11	1.00	-	1.00	-
Yes	166	43629	3.80	3.43	(2.84,4.15)***	1.49	(1.19,1.86)***
CAD							
No	463	313188	1.48	1.00	-		
Yes	4	1800	2.22	1.50	(0.56,4.02)		
CKD							
No	463	313719	1.48	1.00	-		
Yes	4	1269	3.15	2.18	(0.81,5.84)		
COPD							
No	410	293826	1.40	1.00	-	1.00	-
Yes	57	21162	2.69	1.93	(1.46,2.55)***	0.87	(0.65,1.16)
Alcohol-related illness							
No	444	307251	1.45	1.00	-	1.00	-
Yes	23	7737	2.97	2.12	(1.39,3.22)***	1.36	(0.89,2.07)
HIV							
No	467	314394	1.49	1.00	-		
Yes	0	594	0.00	0.00	(0,Inf)		
Medication							
a-blocker							
No	265	286129	0.93	1.00	-	1.00	-
Yes	202	28859	7.00	7.49	(6.24,9)***	4.51	(3.62,5.64)***
b-blocker							
No	406	298298	1.36	1.00	-	1.00	-
Yes	61	16690	3.65	2.63	(2.01,3.45)***	1.22	(0.9,1.65)
CCB							
No	328	271942	1.21	1.00	-	1.00	-
Yes	139	43046	3.23	2.64	(2.16,3.22)***	0.97	(0.74,1.29)
Diuretics							
No	397	289830	1.37	1.00	-	1.00	-
Yes	70	25158	2.78	2.01	(1.55,2.59)***	0.66	(0.49,0.89)**

*: p-value<0.05; **: p-value<0.01; ***: p-value<0.001;

PY: person-years; IR: incidence rate per 1000 person-years; cHR: crude hazard ratio; aHR: adjusted hazard ratio;

CAD: cardiovascular disease; CKD: chronic kidney disease; COPD: chronic obstructive pulmonary disease; HIV: human immunodeficiency virus; AID: Autoimmune disease; CCB: calcium channel blocker; ‡: adjusted by age and hypertension, diabetes mellitus, hyperlipidemia, COPD, alcohol-related illness and all medication.

Supplement Table S3. Baseline characteristic in negative control analysis

	Non-NTS (N=3240)		NTS (N=810)		
Variables	n	%	n	%	p-value
Age, year					0.83
18-30	631	19%	162	20%	
31-40	630	19%	167	21%	
41-50	565	17%	136	17%	
>=51	1414	44%	345	43%	
mean, (SD)	48.1	(18.3)	48.0	(18.8)	0.89
Comorbidities					
hypertension	1036	32%	256	32%	0.87
diabetes mellitus	539	17%	140	17%	0.70
hyperlipidemia	1070	33%	192	24%	<0.001
Stroke	524	16%	90	11%	<0.001
CAD	105	3%	25	3%	0.91
CKD	118	4%	37	5%	0.26
COPD	702	22%	166	20%	0.50
Alcohol-related illness	218	7%	68	8%	0.11
HIV	60	2%	15	2%	1.00
AID	93	3%	28	3%	0.45
Medication					
a-blocker	999	31%	227	28%	0.13
b-blocker	523	16%	125	15%	0.66
CCB	1463	45%	290	36%	<0.001
diuretics	1043	32%	255	31%	0.73

CAD: cardiovascular disease; CKD: chronic kidney disease; COPD: chronic obstructive pulmonary disease;

HIV: human immunodeficiency virus; AID: Autoimmune disease; CCB: calcium channel blocker.

Supplement Table 4. Negative control analysis (NTS infection as the negative control exposure)

	Erectile dysfunction						
Variables	n	PY	IR	cHR (95% CI)		aHR (95% CI)	
non-NTS	48	20892	2.30	1.00	-	1.00	-
NTS	7	5106	1.37	0.59	(0.27,1.31)	0.69	(0.31,1.53)
Age, year							
18-30	4	5769	0.69	1.00	-	1.00	-

31-40	9	5841	1.54	2.23	(0.69,7.24)	1.59	(0.48,5.27)
41-50	13	4592	2.83	4.16	(1.36,12.76)*	1.88	(0.56,6.3)
>=51	29	9796	2.96	4.38	(1.54,12.49)**	1.22	(0.35,4.31)
Comorbidities							
hypertension							
No	32	18918	1.69	1.00	-	1.00	-
Yes	23	7080	3.25	1.93	(1.13,3.31)*	0.80	(0.38,1.67)
diabetes mellitus							
No	41	22418	1.83	1.00	-	1.00	-
Yes	14	3580	3.91	2.20	(1.2,4.04)*	1.08	(0.54,2.16)
hyperlipidemia							
No	24	18515	1.30	1.00	-	1.00	-
Yes	31	7483	4.14	3.23	(1.89,5.52)***	2.57	(1.41,4.69)**
Stroke							
No	48	23324	2.06	1.00	-		
Yes	7	2674	2.62	1.30	(0.59,2.89)		
CAD							
No	54	25413	2.12	1.00	-		
Yes	1	585	1.71	0.82	(0.11,5.92)		
CKD							
No	54	25385	2.13	1.00	-		
Yes	1	613	1.63	0.79	(0.11,5.72)		
COPD							
No	38	21165	1.80	1.00	-	1.00	-
Yes	17	4833	3.52	1.99	(1.12,3.53)*	1.18	(0.63,2.23)
Alcohol-related illness							
No	50	24415	2.05	1.00	-		
Yes	5	1583	3.16	1.57	(0.63,3.94)		
HIV							
No	54	25596	2.11	1.00	-		
Yes	1	401	2.49	1.17	(0.16,8.46)		
AID							
No	51	25279	2.02	1.00	-	1.00	-
Yes	4	719	5.56	2.82	(1.02,7.79)*	2.98	(1.49,5.98)**

Medication							
a-blocker							
No	22	18739	1.17	1.00	-	1.00	-
Yes	33	7259	4.55	3.96	(2.31,6.79)***	2.99	(1.49,5.98)**
b-blocker							
No	42	21802	1.93	1.00	-		
Yes	13	4196	3.10	1.62	(0.87,3.01)		
CCB							
No	24	15109	1.59	1.00	-	1.00	-
Yes	31	10889	2.85	1.81	(1.06,3.08)*	0.68	(0.34,1.37)
diuretics							
No	25	18265	1.37	1.00	-	1.00	-
Yes	30	7733	3.88	2.86	(1.68,4.87)***	1.80	(0.9,3.57)

*: p-value<0.05; **: p-value<0.01; ***: p-value<0.001;

PY: person-years; IR: incidence rate per 1000 person-years; cHR: crude hazard ratio; aHR: adjusted hazard ratio;

CAD: cardiovascular disease; CKD: chronic kidney disease; COPD: chronic obstruction pulmonary disease; HIV: human immunodeficiency virus; AID: Autoimmune disease; CCB: calcium channel blocker; ł: adjusted by age and hypertension, diabetes mellitus, hyperlipidemia, COPD, AID, a-blocker, CCB and diuretics.
